# Closure of the Bering Strait caused Mid-Pleistocene Transition cooling

**DOI:** 10.1038/s41467-018-07828-0

**Published:** 2018-12-19

**Authors:** Sev Kender, Ana Christina Ravelo, Savannah Worne, George E. A. Swann, Melanie J. Leng, Hirofumi Asahi, Julia Becker, Henrieka Detlef, Ivano W. Aiello, Dyke Andreasen, Ian R. Hall

**Affiliations:** 1 Camborne School of Mines, University of Exeter, Penryn Campus, Penryn, Cornwall TR10 9FE UK; 2 NERC Isotope Geosciences Facilities, British Geological Survey, Keyworth, Nottingham NG12 5GG UK; 3 University of California, Santa Cruz, CA 95064 USA; 4 Centre for Environmental Geochemistry, School of Geography, University of Nottingham, University Park, Nottingham, NG7 2RD UK; 5 Centre for Environmental Geochemistry, School of Biosciences, University of Nottingham, Sutton Bonington Campus, Loughborough, NE12 5RD UK; 6 Center for Advanced Marine Core Research, Kochi University, B200 Monobe, Nankoku, Kochi 783-8502 Japan; 7 Department of Geosciences, State Museum of Natural History, 76133 Karlsruhe, Germany; 8 Department of Geoscience, Aarhus University, Høegh-Guldbergs Gade 2, 8000 Aarhus C, Denmark; 9 Moss Landing Marine Laboratories, 8272 Moss Landing Road, Moss Landing, CA 95039 USA; 10 School of Earth and Ocean Sciences, Cardiff University, Cardiff, CF10 3AT UK

## Abstract

The Mid-Pleistocene Transition (MPT) is characterised by cooling and lengthening glacial cycles from 600–1200 ka, thought to be driven by reductions in glacial CO_2_ in particular from ~900 ka onwards. Reduced high latitude upwelling, a process that retains CO_2_ within the deep ocean over glacials, could have aided drawdown but has so far not been constrained in either hemisphere over the MPT. Here, we find that reduced nutrient upwelling in the Bering Sea, and North Pacific Intermediate Water expansion, coincided with the MPT and became more persistent at ~900 ka. We propose reduced upwelling was controlled by expanding sea ice and North Pacific Intermediate Water formation, which may have been enhanced by closure of the Bering Strait. The regional extent of North Pacific Intermediate Water across the subarctic northwest Pacific would have contributed to lower atmospheric CO_2_ and global cooling during the MPT.

## Abstract (web summary)

The causes of Mid-Pleistocene Transition global cooling 1 million years ago are still unknown. Here, the authors find the subarctic North Pacific became stratified during these glaciations due to closure of the Bering Strait, which would have removed CO_2_ from the atmosphere and caused global cooling.

## Introduction

The Mid-Pleistocene Transition (MPT) is the last major transition in Earth’s climate, and appears as global cooling and glacial lengthening in two phases. Firstly, as gradual sea surface temperature (SST) reduction^1^ and glacial intensification and lengthening (towards quasi 100 ka cycles), from about 1200 ka in SST and deep ocean δ^18^O composite records. Secondly, there was abrupt ice sheet growth^2,3^, and further SST reductions^1^ from Marine Isotope Stage (MIS) 22–23 (at ~900 ka). The absence of noteworthy secular changes in Milankovitch forcing, and the weakness of the 100 ka eccentricity insolation cycle, indicates internal feedbacks within the Earth system rather than orbital forcing alone were the cause of these two phases^2,4^. There is currently no consensus on what internal forcing shifts led to the MPT. Hypotheses have pointed to Southern Ocean dust supply increases from ~1300 ka^5^, the unusual insolation low over Antarctica at ~900 ka^2^, changing North American ice sheet dynamics^4,6^ and sea ice changes^7,8^. A secular decline in atmospheric CO_2_ concentrations has been suggested as a central cause^5,6,9–11^, and a slowdown in Atlantic meridional overturning circulation and North Atlantic Deep Water (NADW) production has led to the idea that there was enhanced CO_2_ transfer from the surface ocean to the deep at ~900 ka^12^. These processes are not mutually exclusive, and could each be associated with a build-up of ice on North America for which there is ample evidence^4^. To sequester CO_2_ in the deep ocean also likely requires stifled nutrient and CO_2_-rich deep water upwelling at high latitudes to reduce outgassing, as proposed for the last glacial;^13,14^ however, although recent records of Southern Ocean biogenic opal suggest glacial reductions in productivity occurred throughout the Pleistocene^15^, there are so far no direct records of high latitude upwelling from either hemisphere with which to evaluate this potentially important mechanism for the MPT glacial CO_2_ reduction.

**Fig. 1:**
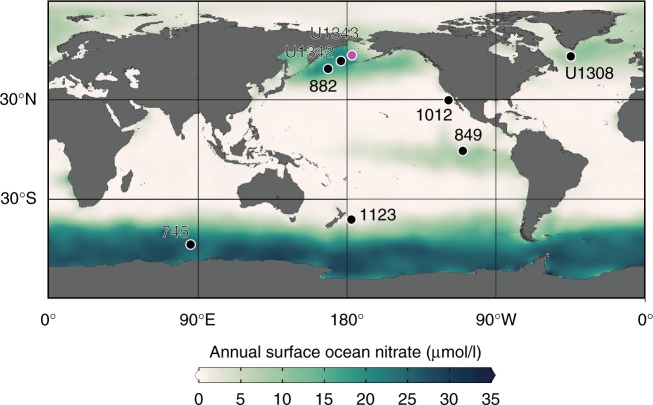
Map of annual surface ocean nitrate concentration (µmol/l). Surface water data taken from the 2013 World Ocean Atlas database^67^. Circles show the locations of sediment cores referred to in the text. The new data in this paper come from Site U1343 (pink circle)

The subarctic North Pacific (Fig. 1), including the marginal Bering Sea, is a high-nutrient, low-chlorophyll (HNLC) region limited by iron, and is in this respect similar to the subantarctic Southern Ocean where major nutrient consumption is incomplete and excess CO_2_ today is vented to the atmosphere^13,16^. The subarctic North Pacific is particularly important, as ventilation of this region may have accounted for 30% of the last deglacial rise in atmospheric CO_2_^17,18^. High-nutrient deep water in the modern subarctic Pacific is separated from the surface by a shallow seasonal halocline. While the stratification is strong enough to prevent major intermediate or deep water formation, nutrient- and CO_2_-rich water sourced from North Pacific Deep Water (NPDW) upwells within eddies along the modern Bering Sea slope (termed the high productivity Green Belt) due to wind forcing, tidal mixing, instability and topographic interference^19,20^. Incomplete nutrient utilization occurs, likely due to iron and/or light limitation^16,21^, leaving surface waters oversaturated compared with atmospheric CO_2_ in the winter^22^. The modern Bering Sea intermediate water mass of low nitrate occurs above the halocline at ~300 m water depth^23^, but eddies along the slope can reach down 400–1000 m^20^.

Glacial reductions in Bering Sea Green Belt primary productivity are similar both to changes in subarctic Pacific primary productivity (ODP Site 882) over at least the last 800 ka^16,21,24,25^, and to glacial reductions in productivity in the Indian Ocean sector of the Southern Ocean (ODP Site 745)^15^, although the causes of reduced productivity are yet to be identified. For example, reductions in Pacific glacial productivity may be caused by light limitation due to a deepening of the mixed layer during summer^24^, hypothetically linked to changing atmospheric weather systems and windiness. Alternatively, changes could be related to reduced glacial upwelling of nutrients^24^ caused by enhanced halocline stratification^16,21^ or the glacial expansion of the nutrient-poor North Pacific Intermediate Water (NPIW) which would have resulted in a less nutrient-rich source of upwelling water^26,27^. Additional possible causes in the Bering Sea are sea ice expansion^8,28^, light limitation^25^, ice-rafted debris^29^, and reduced Fe-fertilization from riverine input and/or remobilization from shelf sediments^19^. A recent MPT productivity record from the Gulf of Alaska does not follow glacial cycles, and has demonstrated fertilization by glacigenic debris as an important process in that region^29^.

In this study we produce the first high latitude nutrient upwelling record over the MPT and determine the causes of reduced glacial productivity in the Bering Sea over MIS 21–29 (840–1020 ka) by presenting new geochemical proxy records from Integrated Ocean Drilling Program (IODP) Site U1343 (1954 m water depth; 175° 49.0′W, 57° 33.4′N), located on a topographic high near the northern Bering Sea slope (Supplementary Fig. 1) within the high productivity Green Belt. We use bulk sediment δ^15^N and δ^13^C of organic carbon (δ^13^C_org_) compared with organic carbon and previously published opal^25^ accumulation rates to decipher changes in deep ocean nutrient supply to the surface. Benthic foraminiferal δ^18^O is used to provide evidence of NPIW expansion. We compare our new records of surface and deep ocean changes with global sea level estimates and published data from other subarctic sites to assess the potential links between cooling, sea ice expansion, closure of the Bering Strait, NPIW production, reduced high latitude CO_2_ and nutrient upwelling, and development of the MPT. We find the increased presence of NPIW, and reduced nutrient upwelling, during glacial periods from the MPT onwards, and propose that coincident Bering Strait closure may have caused NPIW formation by retaining sea ice brines within the Bering Sea.

## Results

### Benthic foraminiferal isotopes

**Fig. 2:**
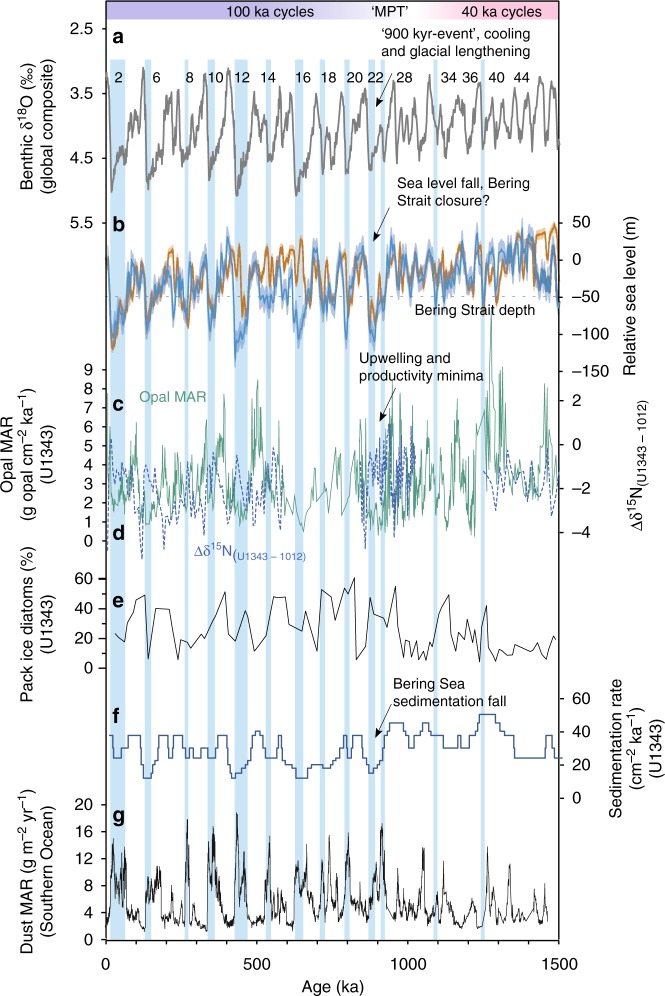
Bering Sea productivity, nutrient utilization and sedimentation rate proxy records compared with global glacial-interglacial cycles for the past 1500 ka. The Mid-Pleistocene Transition (MTP) represents the transition from 40 ka glacial cycles to 100 ka glacial cycles. Major sea level falls below the current Bering Strait sill depth (horizontal dashed line) are indicated with blue vertical bars. From ~900 ka, reduced glacial productivity occurs with increased nutrient utilization. **a** Global benthic foraminiferal δ^18^O composite (grey) LR04^30^. **b** Global sea level estimates from Site 1123 (blue)^2^ and the Mediterranean (orange)^3^. **c** Opal mass accumulation rate (MAR) as a proxy for productivity (green) for Site U1343^25^. **d** Bulk sediment δ^15^N for Site U1343, with Site 1012^48^ subtracted (Δδ^15^N). U1343 δ^15^N data from 850–1020 ka are from this study, other are data are previously published^39,68^. **e** Site U1343 percentage of diatom assemblages regarded as indicative of pack ice^69^. **f** Sedimentation rate (cm ka^−1^) for Site U1343^33^. **g** Dust MAR from the Southern Ocean ODP Site 1090^5^

**Fig. 3:**
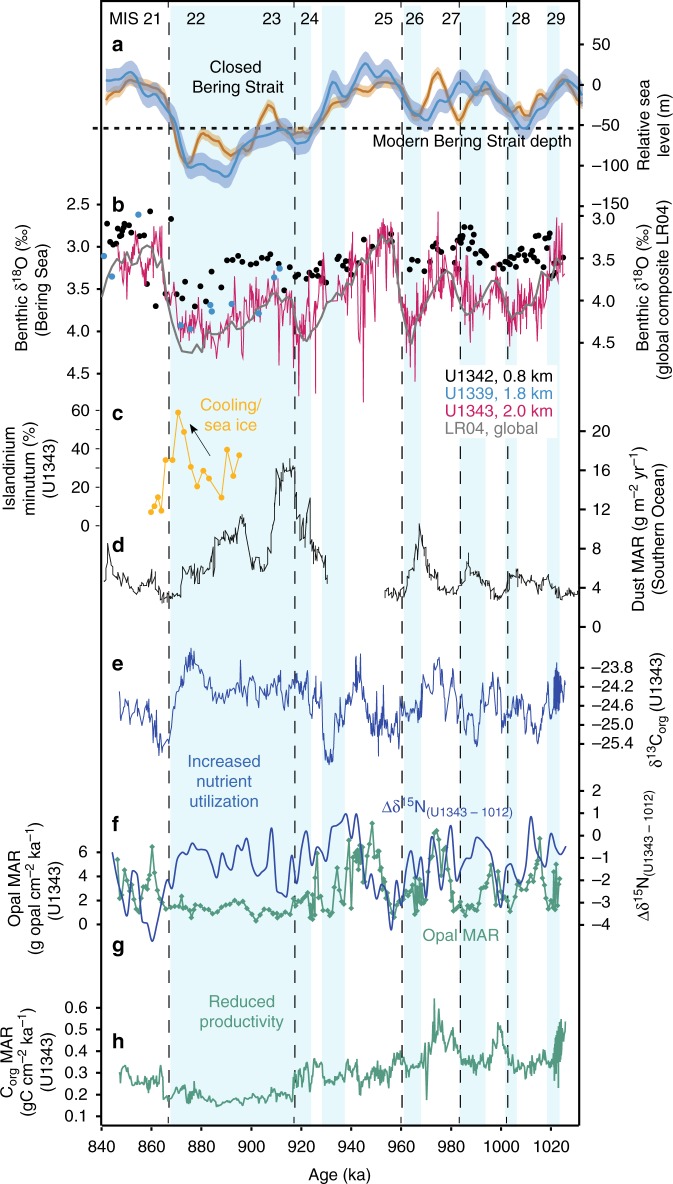
Proxy data from Site U1343 for the time interval 850–1020 ka. Vertical blue bars are times of reduced productivity and elevated nutrient utilization indicating reduced deep water nutrient upwelling along the Bering Slope. **a** Global sea level estimates from Site 1123 (blue)^2^ and the Mediterranean (orange)^3^. The current Bering Strait sill depth is indicated with a horizontal dashed line. **b** Benthic δ^18^O data (this study) from U1343 (red), U1342 (black)^27^, U1339 (blue)^36^ and LR04 (grey)^30^. Data from U1339 and U1342 has been converted to the *Elphidium batialis* scale for direct comparison with U1343 data. **c** Dinoflagellate cyst *Islandinium minutum* % data for U1343 (this study). **d** Dust MAR from the Southern Ocean ODP Site 1090^5^. **e** Bulk sediment δ^13^C_org_ for U1343 (this study). **f** Bulk sediment δ^15^N for Site 1012^48^ subtracted from U1343 (Δδ^15^N). **g** Opal mass accumulation rate (MAR) as a proxy for productivity (green) for Site U1343^25^. **h** Total organic carbon (C_org_) mass accumulation rate (MAR) as a proxy for productivity for Site U1343 (this study)

Our study focuses on the time period ~900 ka (MIS 21–29), which occurs within the MPT when sea levels fell, glacials began to lengthen, and productivity across the Bering Sea and North Pacific fell (Fig. 2a–c; Fig. 3). We produced a composite benthic foraminiferal δ^18^O record at IODP Site U1343 (Fig. 3b) at high temporal resolution (average 200–300 y time step) and used this to create an age model by tuning it to the global composite benthic record LR04^30^ (see Methods). The majority of our benthic δ^18^O record follows long-term trends in LR04 (Fig. 3b, Supplementary Fig. 2a), providing no evidence that long-term changes in water mass occurred at this site (~2 km water depth) throughout almost all of the study interval. However, there is a prolonged negative offset towards relatively low δ^18^O values centred at 880–870 ka during the MIS 22 glacial maximum (Fig. 3b; Supplementary Fig. 2b), and possible offsets at 930 ka although with less certainty (Supplementary Fig. 2c). These offsets toward low δ^18^O values, which are exceptionally clear during MIS 22 at Site U1343, are more typical of shallower water Site U1342 (Fig. 3b). They are important as they contrast from the markedly enriched benthic δ^18^O values in all global records including the eastern equatorial Pacific (Site 849)^31^, deep South Pacific (Site 1123)^32^ and North Atlantic (Site U1308) (Supplementary Fig. 2a).

We are confident the negative MIS 22 δ^18^O offset seen in the Bering Sea records occurred at the glacial maximum (rather than occurring at the beginning of the deglacial) for three reasons. First, to shift the benthic δ^18^O record to better match the global composite deglacial timing would require rapid and drastic changes in sedimentation rates, which are not seen elsewhere at Site U1343^33^. Second, manipulating the age model in this way would create an unusual three-step shape to the deglaciation, not seen in other records including the Pacific^2,31^. Third, our dinoflagellate cyst assemblages indicate that the relatively low δ^18^O values are accompanied by a probable cooling and/or expansion of sea ice (Fig. 3c), indicative of a glacial maximum rather than deglaciation. The interval 870–880 ka is characterised by an increase in the proportion of dinoflagellate cyst *Islandinium minutum* which is a species known to become abundant in the modern Arctic and Atlantic when the temperature is approximately ~0–6 °C, and sea ice is >8 months/year^34^. The other abundant species in our samples, *Bitectatodinium* spp. and *Brigantedinium* spp. (Supplementary Data 1), are cosmopolitan and do not show strong preferences for temperature or sea ice^34^, although *Islandinium* and *Bitectatodinium* are often associated with polynya^35^.

The benthic foraminiferal δ^13^C data show wide variability (Supplementary Fig. 3a). This is likely due to the different species habitats and possible authigenic carbonate overgrowth^36^, although the majority of the species aside from *Elphidium* sit above the −2.5‰ threshold below which is considered indicative of possible authigenic carbonate for the Bering Sea^36^. In order to avoid authigenic carbonates, and the impact of changing species habitats, we plot the Site U1343 δ^13^C of *Uvigerina* spp. which shows overall similar trends to δ^13^C of *Uvigerina* spp. from equatorial Pacific Site 849 with a −1‰ offset (Supplementary Fig. 3b). Although there is scatter in the data, there is evidence for a positive δ^13^C offset of Site U1343 from 849 during the MIS 22 glacial maximum, coinciding with the negative δ^18^O offset of U1343 from the LR04 global stack.

### MPT changes in primary productivity

Obtaining accurate proxies for export production can be a challenge due to preservation issues with both organic and inorganic compounds^24^. Bering Sea Site U1343 is uniquely valuable in this respect, as it sits within the highly productive Green Belt region dominated by diatom production such that the sediment is typically enriched in biogenic opal (the constituent of diatom frustules) at ~5–30%^25^. Because factors such as diagenesis and low opal export may compromise the preservation of opal^21,28^, and any shifts in the balance of phytoplankton towards calcifiers could impact opal accumulation, absolute values may not constitute a quantitative proxy for export production. However, in this high flux region^37^ the relative changes in opal accumulation are large and likely to reflect first-order changes in productivity. Site U1343 constitutes a continuous long record that has been orbitally-tuned using δ^18^O stratigraphy over the past 1.2 Ma^33^ allowing glacial-interglacial calculation of sedimentation and biogenic opal accumulation rates. As the age model is constructed with a relatively small number of tie points (see Methods), the resulting opal and total organic carbon mass accumulation rates (MARs) are likely to approximate true flux rates only on long glacial-interglacial timescales. The adjustment of opal and total organic carbon percentages to MAR does not change the overall pattern in a significant way, although it does lower the glacial MAR to some extent (Supplementary Fig. 4). Figure 2c shows that long term opal changes over the past 1.5 Ma exhibit highly fluctuating values, with generally higher accumulation rates before 900 ka, and higher rates during interglacials overall^25^.

We generated a high resolution (average 200–300 yr time step) record of organic carbon (C_org_) accumulation over the interval 840–1020 ka at Site U1343 (Fig. 3h, Supplementary Fig. 4), which is subject to different limitations to biogenic opal but is complementary as a productivity proxy^37^. Sedimentary C_org_ accumulation shows first-order trends similar to those found in the opal accumulation record (Fig. 3g), with cyclic higher values before 920 ka, and lowest values during MIS 22–23 thus lending confidence that opal accumulation at U1343 is indicative of export production. The more extreme decrease in C_org_ accumulation at ~920 ka compared to opal accumulation could be due either to reduced C_org_ preservation in comparison to opal^38^, a shift away from calcareous phytoplankton towards siliceous, or changes to phytoplankton Si/C and Si/N uptake ratios due to changing Fe availability^39^.

### Surface nutrient supply and upwelling

We generated bulk sediment δ^15^N (Supplementary Fig. 2e) and δ^13^C_org_ (Fig. 3e) to provide insight into surface water nutrient utilization and possible causes for the observed changes in productivity. In some subarctic settings it is known that a significant proportion of total N can be bound up in inorganic form^40^, but we do not believe this significantly affects our samples as total N versus total C_org_ shows no positive intercept on the total N axis (Supplementary Fig. 5). Although phytoplankton preferentially take up ^14^N nitrate^41^, variations in sediment δ^15^N are controlled by a variety of factors: changes in the δ^15^N of nitrate in advected and/or upwelled source water (δ^15^N_source_), which can be impacted by local or regional water column denitrification (e.g., denitrification enriches the δ^15^N_source_);^42^ nitrate utilization during marine organic production (e.g., higher utilization of available nitrate results in higher δ^15^N of exported organic matter);^42^ diagenetic alteration;^43^ and contributions of terrestrial organic material which has lower δ^15^N (~3 ‰)^44^ and δ^13^C_org_ (–26‰)^45^ values than marine organics. Diagenesis is unlikely a major influence in the high sedimentation rate and productive Bering Sea^21,46,47^. Although it is possible that increased terrestrial material caused low δ^15^N and δ^13^C_org_ during low export production intervals before 930 ka, we regard this as unlikely due to the relatively stable sediment accumulation rates from 920–1020 ka, and relatively low C/N values (indicative of a low terrestrial:marine ratio) that do not co-vary with δ^15^N or δ^13^C_org_ (Supplementary Fig. 6). Thus, observed variations in δ^15^N at U1343 likely reflect nitrate utilization and/or changes in water column denitrification affecting δ^15^N_source_.

From ~930–1020 ka (MIS 25–29) both opal accumulation and δ^15^N exhibit significant cyclicity at the 28 ka band, with opal leading δ^15^N (Supplementary Fig. 7b). In the latter part of the cycles, reductions in opal accumulation are coincident with sustained high δ^15^N (blue bars in Fig. 3 and Supplementary Fig. 7a). This suggests these times were characterised by lower productivity and elevated nutrient utilization, which can be explained by reduced upwelling of nutrient-rich deep water but sustained utilization from continued iron delivery^21^. To account for possible changes in the δ^15^N_source_ of surface and/or subsurface water imported into the Bering Sea from the northeast Pacific (via the Alaskan Stream^28^), from our δ^15^N record we subtract eastern North Pacific Site 1012^48^ (Supplementary Fig. 2d–f). Although this site is thought to be subject to intense denitrification^48^, it is considered to represent some component of the regional changes in δ^15^N_source_ due to its similarity to δ^15^N records of northeast Pacific sites over more recent glacial cycles^49^. The resulting Δδ^15^N_U1343–1012_ (Fig. 3f, Supplementary Fig. 7) shows broadly similar trends to δ^15^N between 930–1020 ka, supporting the observation that nutrient utilization (high δ^15^N) is lagging productivity (opal). The lag is more pronounced in Δδ^15^N than δ^15^N, which may be real or possibly due to an issue with age model uncertainty of the two sites. The 28 ka cyclicity disappears after 930 ka, and Δδ^15^N remains high and productivity (opal and C_org_ accumulation) low for the entirety of MIS 22–23 (Fig. 3f, g) signifying a prolonged reduction in upwelling nutrients.

The Δδ^15^N_U1343–1012_ nutrient utilization proxy for U1343 is potentially impacted by the low-resolution nature of the Site 1012 record, by possible discrepancies between age models at both sites, and by possible denitrification. Although glacials have been considered times of reduced denitrification in the Bering Sea and subarctic North Pacific due to lower export production^21^, peaks in Bering Sea δ^15^N during deglacial oxygen minima have been proposed as possible denitrification signals^21^. To test this we compare the Δδ^15^N_U1343–1012_ to the Site U1343 δ^13^C_org_ (Fig. 3e) which is controlled by nutrient utilization, although also impacted by changes in surface water CO_2_ and plankton community structure. Despite these potential limitations, for the majority of the record the major, long-term changes in δ^13^C_org_ support the Δδ^15^N_U1343–1012_ findings of cyclic changes before about 930 ka, higher nutrient utilization (removal of the bioavailable ^12^C) throughout the entirety of MIS 22–23, with highest values during MIS 22 before a rapid return to low nutrient utilization during interglacial MIS 21 (Fig. 3e).

## Discussion

**Fig. 4:**
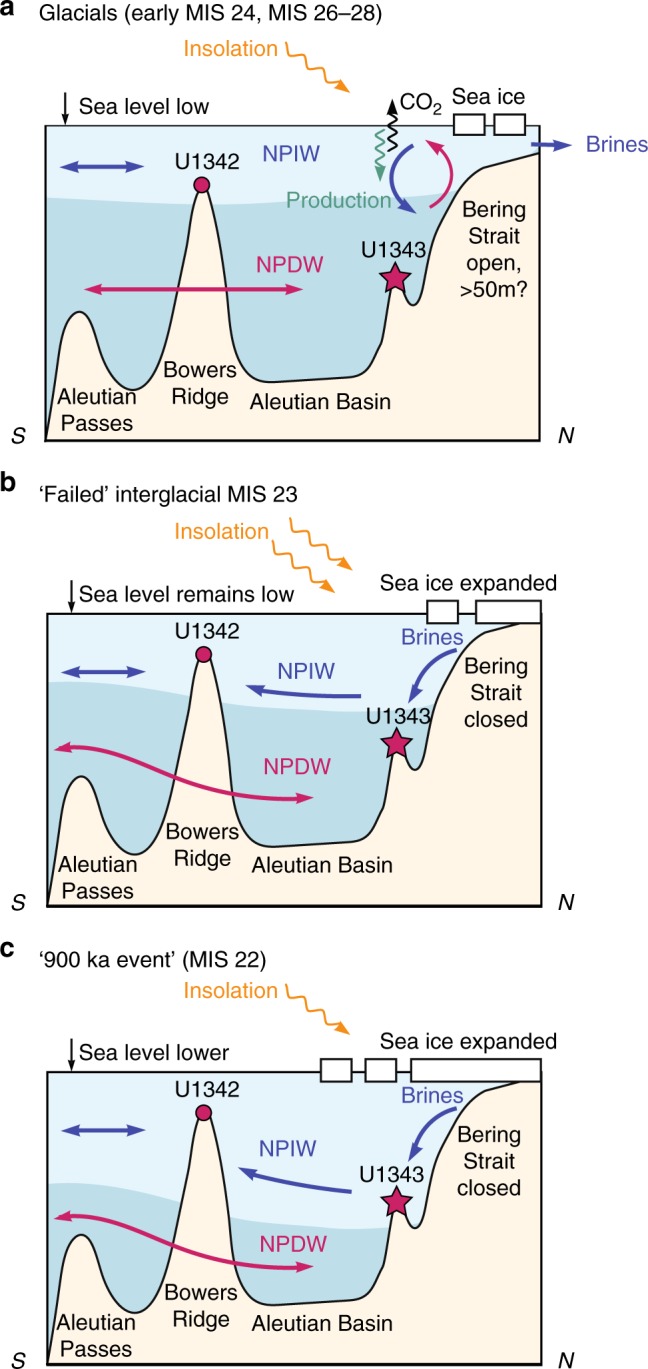
Schematic Bering Sea cross section (N-S) showing palaeoceanographic interpretations. **a** Glacials between MIS 24–28 (before 930 ka). **b** ‘Failed’ interglacial MIS 23. **c** Glacial MIS 22 (the ‘900 ka event’). Interpretations of North Pacific Intermediate Water (NPIW) and North Pacific Deep Water (NPDW) are based on benthic δ^18^O from Sites U1343 (this study) and U1342^27^. Arrows show the inferred flow of warmer (red) and colder (blue) currents

The reductions in nutrient upwelling (high Δδ^15^N and lower opal accumulation; blue bars in Fig. 3) between 930–1020 ka are predominantly restricted to glacial intervals between MIS 24–28, with nutrient upwelling and productivity increasing after each deglacial (dashed black lines in Fig. 3). Parts of this interval are characterised by covariation of utilization and productivity, which could be explained by variations in iron fertilization, perhaps from terrestrial sources to the region. Conversely, when productivity decreases and nutrient utilization increases (blue bars in Fig. 3), enhanced nutrient utilization cannot be accounted for by, for example, iron fertilization as this would increase productivity. Enhanced halocline stratification is unlikely to have caused these productivity minima along the Bering Slope (unlike at Bowers Ridge^21,47^) as the modern Pacific halocline is relatively shallow (upper 300 m) and eddies at the slope can reach down to 1 km water depth^20^. These strong eddies are also likely to rule out a change in the depth of the shallower mixed layer as the primary cause of reduced productivity. We propose the cause of the reduced glacial nutrient upwelling from 930–1020 ka was due to enhanced NPIW (Fig. 4a). NPIW today produces a temperature and salinity low centred at ~500 m in the North Pacific, which is sourced partly from winter Okhotsk Sea pack ice brine rejection, and exhibits low nutrients including nitrate^50^. Evidence for expanded NPIW during the last glacial maximum is extensive^26,27,36,51,52^, with *ɛ*_Nd_ records identifying the Bering Sea as a probable source region for sea ice brine rejection during glacials^26,52^. The central Bering Sea appears to have been bathed in a relatively nutrient-poor (likely younger than deep Pacific) NPIW to at least ~0.8 km water depth during glacials over the past 1.2 Ma, evidenced by Site U1342 (Bowers Ridge) negative offsets in benthic foraminiferal δ^18^O from deep Pacific end members^27^. These records indicate that the lower boundary of NPIW deepened to >0.8 km water depth during glacials^27^, offsetting shallow benthic δ^18^O from U1343 values at ~2 km water depth (Fig. 3b). The proposed mechanism for producing relatively low Bering Sea deep water δ^18^O is sea ice brine rejection, which can increase salinity without significantly altering δ^18^O, and is able to transport highly depleted δ^18^O surface water to depth^27,36^.

**Fig. 5:**
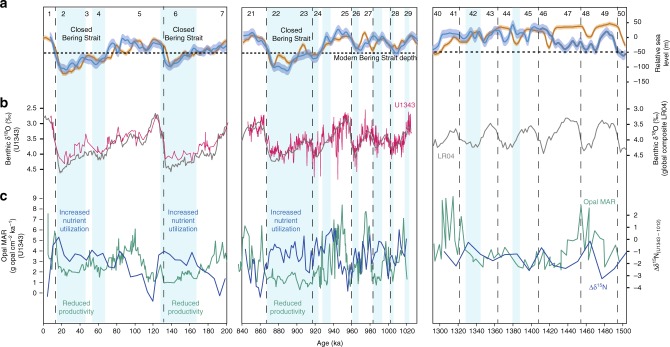
Proxy data from Site U1343 compared with sea level, for three time periods over the past 1.5 Ma. Vertical blue bars are times of reduced productivity and elevated nutrient utilization indicating reduced deep water nutrient upwelling along the Bering Slope. **a** Global sea level estimates from Site 1123 (blue)^2^ and the Mediterranean (orange)^3^. The current Bering Strait sill depth is indicated with a horizontal dashed line. **b** Benthic δ^18^O data from U1343 (pink) and LR04 (grey)^30^. U1343 δ^18^O data from 850–1020 ka are from this study, other data are published elsewhere^33^. **c** Bulk sediment δ^15^N for Site 1012^48^ subtracted from U1343 (blue) (Δδ^15^N). U1343 δ^15^N data from 850–1020 ka are from this study, other are data are published elsewhere^39,68^. Opal mass accumulation rate (MAR) as a proxy for productivity (green) for Site U1343^25^

The shift away from precession (~23–28 ka) frequency in opal accumulation cycles at ~930 ka (Fig. 2c, Supplementary Fig. 8), suggests a change in the causal mechanisms driving export production during the MPT, possibly from a combination of NPIW and terrigenous nutrient influences before 930 ka, towards predominant NPIW control thereafter. During MIS 24–22, reduced nutrient upwelling (increased nutrient utilization and lower productivity) occurred for an extended period of time (>30 ka). This striking feature of the dataset is similar to δ^15^N and opal accumulation records during the last glacial from Bowers Ridge (low glacial opal accumulation and higher δ^15^N)^21^, and the last glacial cycle from the Bering Slope (Fig. 5). This provides evidence for both a more limited upwelling nutrient supply, and more complete nitrate utilization from continued iron delivery, during severe glacials^21^. During the ‘failed’ interglacial MIS 23, which directly contributed to the first quasi 100-ka cycle, nutrient upwelling remained low (blue bars in Fig. 3) likely due to the sustained expansion of NPIW, whilst insolation at 65°N was increasing (Fig. 4b). Subsequently, MIS 22 exhibited a further deepening of the lower boundary of NPIW to >2 km water depth, as evidenced by U1343 offsets in benthic δ^18^O towards shallower water values (Fig. 3b) and potentially δ^13^C (Supplementary Fig. 3) from global and Pacific records respectively, further removing the NPDW source of nutrients from the photic zone, and stifling both productivity and CO_2_ outgassing (Fig. 4c). A lower resolution record from Site U1339 at 1.8 km water depth is consistent with this hypothesis, with benthic δ^18^O values similar to Site U1343 (Fig. 3b). When considering the long-term record of nutrient utilization and primary productivity at Site U1343 (Figs. 1c, d, 5c), the data available show that severe glacials from MIS 22–24 onwards (MIS 2, 6, 8, 12 and 14) were coincident with longer productivity crashes and reduced nutrient upwelling (blue bars in Fig. 5). There is also evidence for deep expansion of NPIW to greater that 2 km from the lower resolution benthic δ^18^O during these latter glacials (Fig. 5b). However, before ~930 ka productivity and nutrient utilization proxies indicate shorter-lived times of reduced nutrient upwelling, which we propose was due to less influence of NPIW in the early part of the MPT and before.

A cause for expanded NPIW has been suggested as enhanced sea ice and the production of brines, perhaps due to shifts in wind stress promoting polynya formation near the shelf^27^. Modelling of wind patterns over the last glacial does show significantly increased windiness over the Bering Sea^18^. A sea-ice controlled expansion of NPIW during glacials over the MPT is consistent with increased proportions of diatoms at the Bering Slope indicative of pack ice (Fig. 2e), although these records are of low resolution. Higher resolution but discontinuous IP_25_ and HBI III proxy records from U1343 were interpreted as glacial expansion of seasonal sea ice from 1150 ka, caused by high latitude atmospheric cooling^1^, and then a more persistent seasonal/extended sea ice cover during glacials from MIS 22, possibly from reduced Pacific surface water influence^8^. Our dinoflagellate cyst assemblage record is consistent with an MIS 22 expansion of sea ice (Fig. 4c). Although a continuous and high-resolution sea ice proxy record from the Bering Sea is yet to be produced, further evidence for a sea ice control on NPIW and Bering Slope nutrient upwelling comes from sedimentation rate reductions along the slope during glacials particularly from MIS 22–24 onwards (Fig. 2f). Enhanced glaciation around the Bering Sea would have expanded shorefast and pack ice, known to inhibit modern day winter sedimentation^53^.

Glacial expansions of sea ice influence may have been controlled by reduced air temperatures and/or increased windiness, and another factor promoting these conditions could have been closure of the Bering Strait^27^. Modern polynya brines formed in the Gulf of Anadyr and Anadyr Strait region of the Bering Sea flow into the Arctic^54^. A closed Bering Strait would have retained glacial brines within the Bering Sea. Modelling studies of a closed Bering Strait^55^, although failing to predict expanded glacial NPIW perhaps due to limited modelling of Bering Sea shelf processes^27^, do show entrained colder water within the Bering Sea. The first marked long-term period of reduced nutrient upwelling in our records was at MIS 22–24, which coincides with the first time sea level was below 50 m of the present^2,3^ (Fig. 2b). The modern Bering Strait sill depth is 50 m. This region is thought to have undergone subsidence in the Pliocene^56^ and first opened at about 4.8–5.5 Ma^57^. After an initial period of southward flow, the strait has been exchanging water with the Arctic in a (modern) northward flow since 3.3 Ma^58,59^. There is no direct evidence for how the water depth of the Bering Strait may have changed since, and it is possible that the strong currents did not allow significant sediment deposition and therefore any significant change from its current depth of ~50 m. We hypothesise that the failed MIS 23 deglacial may have directly coincided with the first closure of the Bering Strait (and subsequent closure during long glacials thereafter; Fig. 5a), retaining brines and NPIW in the Bering Sea, and cutting off the source of deep nutrients and CO_2_ from the photic zone (Fig. 4b, c). This would have acted as a positive feedback on a process of global cooling already in motion. In addition to lower glacial temperatures and increased windiness, the idea of a closed Bering Strait promoting further enhanced sea ice, brines, NPIW and reduced nutrient upwelling along the Bering Slope, can only be supported by a comparison of upwelling with sea level records. Over the last 1.5 Ma, nutrient upwelling (offset between opal MAR and Δδ^15^N) has been lowest when sea level^2^ was below 50 m of present (Table 1), compared to when global SST was below –3 °C of present, or deep ocean δ^18^O (combined deep ocean temperature and ice volume) was above 4‰ (severe glacials). This provides some support for a closed Bering Strait having a stronger influence on NPIW and nutrient upwelling in the Bering Sea than either the average global surface or bottom water temperatures alone.

**Table 1:** Average nutrient upwelling for glacial and interglacial phases

Proxy	‘Midpoint’	Opal MAR–Δδ^15^N: warmer than midpoint	Opal MAR–Δδ^15^N: cooler than midpoint
Sea level^2^	–50 m	5.91	3.66
Global SST^70^	–3 ^o^C	6.11	4.68
Deep ocean δ^18^O (LR04)^30^	+4‰	6.54	4.38

Average extent of nutrient upwelling (opal MAR–Δδ^15^N) for different proxies, for both above and below their 1.5 Ma approximate glacial ‘midpoint’ values (for the dataset available, Fig. 2). For sea level^2^, –50 m from modern is chosen as the current depth of the Bering Strait. For global sea surface temperature^70^ (SST), –3 ^o^C from modern is chosen as –6 ^o^C was the last glacial maximum. For global benthic δ^18^O (ref. 30), + 4‰ is the midpoint between the ~3–5‰ glacial-interglacial range. The lowest value of average nutrient upwelling (opal MAR–Δδ^15^N) is when sea level is below 50 m of present.

The regional significance of reduced MPT nutrient upwelling along the Bering Slope, caused by NPIW expansion (Fig. 5), is that NPIW would have inhibited CO_2_ atmospheric exchange not only in the Bering Sea, but also across the subarctic North Pacific^13,16^, creating a wide regional barrier for the upwelling of CO_2_-rich deep water and associated primary productivity. Recent studies have highlighted the importance of North Pacific deep overturning^60^ for increased outgassing of deep ocean CO_2_ during the last deglacial^17,18^, and the role that expanded NPIW may have played in suppressing North Pacific CO_2_ ventilation to the atmosphere during the last glacial maximum^18^. A closed Bering Strait, therefore, could have contributed to MPT cooling by promoting local sea ice^8^ and regionally-extensive NPIW and reduced nutrient upwelling (this study). NPIW would have created an effective barrier to deep North Pacific overturning, and subsequent atmospheric ventilation of CO_2_. In this scenario, overturning of the North Pacific from the MPT onwards was only instigated at the termination of severe glacial conditions and associated global ocean circulation changes such as a shutdown of Atlantic Meridional Overturning Circulation^60^.

The initial cause of MPT cooling remains enigmatic, but our new records show the North Pacific and the Bering Strait were important components within this global climate shift. Atmospheric cooling in high latitudes began at ~1.2 Ma^1^ and may have been associated with a reduction in atmospheric CO_2_ from enhanced dust fertilization in the Southern Ocean^5^ (Fig. 2g). There is evidence for increases in Bering Sea sea ice from at least 1.2 Ma^8^, and possible associated expansion of NPIW from reduced primary productivity at 1.2 Ma (Fig. 2c). These pieces of evidence suggest both the North Pacific and the Southern Ocean were likely ventilating less CO_2_ to the atmosphere during glacials from the early phase of the MPT, although further records are needed to test this and ascertain timings. The second step in the development of the MPT, the severe ice sheet expansion^3^ and glacial lengthening at ~900 ka (MIS 22–24; Fig. 2a), has been suggested to be associated with further CO_2_ decline from deep ocean reorganisation and sequestration of CO_2_ (ref. ^12^). In agreement with this, we find that MIS 22–24 was associated with a severe and extended period of reduced Bering Slope nutrient upwelling and NPIW expansion to >2 km water depth, similar to subsequent severe glacials (Fig. 5), which would have further inhibited any CO_2_ ventilation across the subarctic North Pacific. We suggest closure of the Bering Strait was contributory to this expanded NPIW, either as a threshold response crossed due to an as yet undefined boundary condition change, or as a boundary condition change in itself such as a hypothetical shoaling of the strait through sediment accumulation. This could be tested with future coring of the Bering Strait to ascertain its sedimentation rate history. Southern Ocean productivity proxies are currently of low resolution^15^, and although they show consistently increased glacial water column stratification, thought critical for sequestration of glacial CO_2_ (ref. ^14^), they do not show a clear increase in stratification over the MPT^15^ highlighting the potential importance of NPIW in increased oceanic sequestration of CO_2_ during the MPT. Although a further enhancement of dust deposition across the Southern Ocean may have contributed to MIS 22–24 cooling and CO_2_ decline (Fig. 2g), we note that dust deposition was actually decreasing into the MIS 22 glacial maximum (Fig. 3d) whilst nutrient upwelling on the Bering Slope remained inhibited and NPIW expanded, thus supporting changes in upwelling of the North Pacific as an important mechanism of CO_2_ reduction compared with Southern Ocean dust fertilization.

The cause of MPT glacial lengthening is still unknown, but it may have emerged from the ‘skipping’ of insolation peaks^61–63^ ultimately due to lower glacial CO_2_ associated with oceanic processes described here. The lower global temperature may have been able to sustain larger ice volumes that could withstand melting and override these skipped insolation peaks. In this mid-late Pleistocene state, abrupt deglacials were likely triggered by a breakdown in Southern Ocean stratification and enhanced CO_2_ ventilation^14^, followed by reduced NPIW in the North Pacific and further enhanced CO_2_ ventilation^18^. Our records cannot address the boundary condition changes at ~1.2 Ma that instigated global cooling, which could have been from changing ice sheet dynamics described in the ‘regolith hypothesis’^4^. However, they do suggest that enhanced glacial drawdown of CO_2_ occurred during the MPT and in particular from ~900 ka (MIS 22–23), due at least in part to NPIW expansion across the subarctic North Pacific and associated reduced upwelling. Further high-resolution continuous sea ice and upwelling records over the last 1.5 Ma, from both the subarctic North Pacific and the Southern Ocean, and numerical modelling studies with integrated shelf processes, are now needed to explore the full climatic impacts of a closed Bering Strait and development of the MPT.

## Methods

### Site U1343

Surface water in the Bering Sea enters from the North Pacific, primarily from the relatively warm Alaskan Stream (Supplementary Fig. 1a)^20^. Deep water that bathes Site U1343 is fed from NPDW, which enters and exits the Bering Sea through various passes in the Aleutian Island Arc (sill depths <4000 m). The clastic sediments from Site U1343 are predominantly fine clay and biogenic material^64^. There are no transported shelf-characteristic benthic foraminifera recorded^65^, which is to be expected as Site U1343 is situated on a topographic high separated from the shelf (Supplementary Fig. 1b), and is at ~1954 m depth. There were no obvious current-formed sedimentary features recorded by shipboard sedimentologists. Bioturbation is recorded as centimetre scale mottling of ash layers and gradational lithological transitions, and no long bioturbation features (e.g., burrows) were recorded^65^. Therefore, our maximum sampling frequency of 8 cm (ave. ~250 yrs) was designed to override the major effects of bioturbation. We sampled between 219.87 and 281.74 m composite core depth (m CCSF-A). Site U1343 was double piston-cored to ~262 m CCSF, and the constructed splice leaves no apparent coring gaps in this section^65^.

### Foraminiferal isotopes

For δ^18^O and δ^13^C of benthic foraminifera (Supplementary Data 2), bulk samples were freeze-dried, weighed, processed through a 63 μm sieve with de-ionised water, and oven dried at <40 °C. Analysis was carried out on a range of benthic foraminiferal species (Supplementary Fig. 9), focusing on *Elphidium batialis* or *Uvigerina bifurcata* wherever abundant specimens were available. We measured ~830 benthic foraminiferal samples. For each sample, ~5 well-preserved shells were selected from the >150–250 μm fraction. Analyses were performed using a Finnigan MAT252 mass spectrometer with automated carbonate preparation device at Cardiff University stable isotope facility, and an IsoPrime mass spectrometer with a Multicarb preparation system at the NERC Isotope Geosciences Laboratory at the British Geological Survey. Stable isotope results were calibrated to the VPDB scale by international standards. The analytical precision is better than ± 0.08‰ at Cardiff, and ± 0.05‰ at NIGL.

### Bulk sediment geochemistry

We measured ~740 samples for total organic carbon (TOC) and isotopes of C_org_ (δ^13^C_org_) (Supplementary Data 3). Around 500 mg sample was added to 5% HCl to remove calcite, rinsed in deionized water, dried at 40 °C, ground to a fine powder and homogenized. The samples were combusted using a Costech ECS4010 elemental analyser at approximately 1050 °C, and then analysed using a VG Optima dual inlet mass spectrometer at the NERC Isotope Geosciences Laboratory (British Geological Survey). δ^13^C_org_ is expressed as per mil (‰) in conventional delta notation relative to the ^13^C/^12^C ratio calculated to the Vienna Pee Dee Belemnite (VPDB) scale, utilising within-run laboratory and international standards. Analytical reproducibility for the within-run standards was <0.1‰ for δ^13^C, and <0.7% for TOC.

For δ^15^N, ~215 bulk sediment samples (unacidified) were run on a Carlo Erba 1108 elemental analyzer, interfaced to a Thermo Finnigan Delta Plus XP IRMS at the University of California, Santa Cruz (Supplementary Data 4). δ^15^N is expressed as per mil (‰) in conventional delta notation relative to atmospheric nitrogen. Precision for δ^15^N is 0.2‰, based on replicates and in-house sediment standards.

Total organic carbon (TOC) mass accumulation rate (C_org_ MAR) was calculated as (dry bulk density) × (sedimentation rate) × (wt %TOC). Opal MAR was calculated as (dry bulk density) × (sedimentation rate) × (wt %opal). Measured dry bulk density was only carried out on U1343 Hole A, and the bottom part of Hole E (ref. ^65^). We therefore estimated dry bulk density for the U1343 splice by converting the depths to composite m CCSF values, and modelling a 9-point loess smoothing spline for the entire record. We then estimated sample dry bulk density values to the nearest 10 m (by linear interpolation).

### Age model

We constructed a composite benthic foraminiferal δ^18^O record to provide a more precise age model for this interval than previously provided by δ^18^O stratigraphy^33^ (Supplementary Fig. 10). With just five age-depth tie points (triangles in Supplementary Fig. 2, Supplementary Table 1) based on major deglacials, we obtain a good match (*r*^2^ = 0.78, Supplementary Fig. 11) between U1343 and the global composite stack^30^ (LR04) when our record is smoothed, with a consistent offset of ~0.5‰. This correlation suggests no significant missing intervals, as is expected with high sedimentation rates at U1343. Although our age model is markedly more precise than previously reported for U1343, when comparing our records with other locations it should be noted that up to 10^3^ yr offsets exist between δ^18^O records in different ocean basins^33^.

To estimate offsets between species and construct the composite benthic foraminiferal δ^18^O record, we firstly removed all δ^18^O data points where the values for δ^13^C were below –5‰ (signifying potential diagenetic alteration), and removed all significantly high/low δ^18^O data points if there was a replicate measurement for that sample. We then calculated offsets between all species compared with *Elphidium* (Supplementary Figs 9, 12, Supplementary Table 2). We defined the *Elphidium-Uvigerina* offset by combining all our paired *Elphidium*/*Uvigerina* data with previously published data from U1343^33^. This produced an *Elphidium-Uvigerina* offset of 0.87 ± 0.2‰. We applied this, as we found the offsets between *U. senticosa* and *U. bifurcata* were not outside of this range, and prefer to use the more robust offset calculated from the larger dataset. We note that our value is slightly larger than the offset provided by Asahi et al. (ref. ^33^). We used the previously defined offsets for *Globobulimina pacifica* (0.85 ± 0.22‰) and *Nonionella labradorica* (0.65 ± 0.27‰) from U1343^33^, as our data were collected on a much lower number of samples and produced similar offset values (0.86 and 0.62‰, respectively). To estimate offsets for the other species, we calculated a smoothing spline because we did not have paired measurements in enough samples. The offsets for the species *Islandiella norcrossi*, *Valvulineria aurucana*, *Lenticulina* spp. and *Cassidulina* spp. were estimated by subtracting their values from the smoothing spline value at that location (Supplementary Table 2).

### Dinoflagellate cysts

Palynological samples were chemically treated following the procedure used at GEOTOP^66^. Briefly, 5 cc of wet sediment were sieved through 10 and 106 µm to discard coarse sand and silt, and fine clay. The fraction between 106 and 10 µm was then treated with warm HCL (10%) and HF (49%) several times to dissolve carbonate, silicate, and aluminosilicates. The residue was finally mounted between a slide and cover slide in glycerin gel. Organic-walled dinoflagellate cysts were counted using an optical microscope at magnification of ×400 to ×1000 . At least 300 dinoflagellate cysts were counted for each sample when possible, otherwise the entire slide was scanned and the average count is 214 cysts (Supplementary Data 1).

## Supplementary information

Supplementary Information

Description of Additional Supplementary Files

Supplementary Data 1

Supplementary Data 2

Supplementary Data 3

Supplementary Data 4

## Data Availability

All data generated during this study supporting its findings are available within the paper and the supplementary information.
