# Supplementary material for: Closure of the Bering Strait caused Mid-Pleistocene Transition cooling
Source: Nat Commun. 2018 Dec 19;9:5386. doi: 10.1038/s41467-018-07828-0 (PMC6300599; doi:10.1038/s41467-018-07828-0)
Supplement: Supplementary file 1 — Supplementary Information [file 41467_2018_7828_MOESM1_ESM.pdf]

Supplementary Information

Closure of the Bering Strait caused Mid-Pleistocene Transition cooling

Kender et al.

Contents:

Supplementary Fig. 1. Location maps.

Supplementary Fig. 2. Stable isotope offsets between sites.

Supplementary Fig. 3. Foraminiferal isotope data compared with published datasets.

Supplementary Fig. 4. Comparison between % and MAR of proxies

Supplementary Fig. 5. Total nitrogen against total organic carbon.

Supplementary Fig. 6. Bulk sediment C/N ratios.

Supplementary Fig. 7. Spectral analysis of $\delta^{15}N$ and opal accumulation.

Supplementary Fig. 8. Spectra and wavelet analyses for IODP Site U1343.

Supplementary Fig. 9. Raw and composite benthic foraminiferal $\delta^{18}O$.

Supplementary Fig. 10. Benthic $\delta^{18}O$ data from Site U1343 aside global composite stack LR04.

Supplementary Fig. 11. Benthic $\delta^{18}O$ data between 840–1020 ka vs. LR04.

Supplementary Fig. 12. Benthic foraminiferal species $\delta^{18}O$ vs. *Elphidium* $\delta^{18}O$.

Supplementary Table 1. Age control points for U1343.

Supplementary Table 2. Mean species offsets of $\delta^{18}O$ for Site U1343.

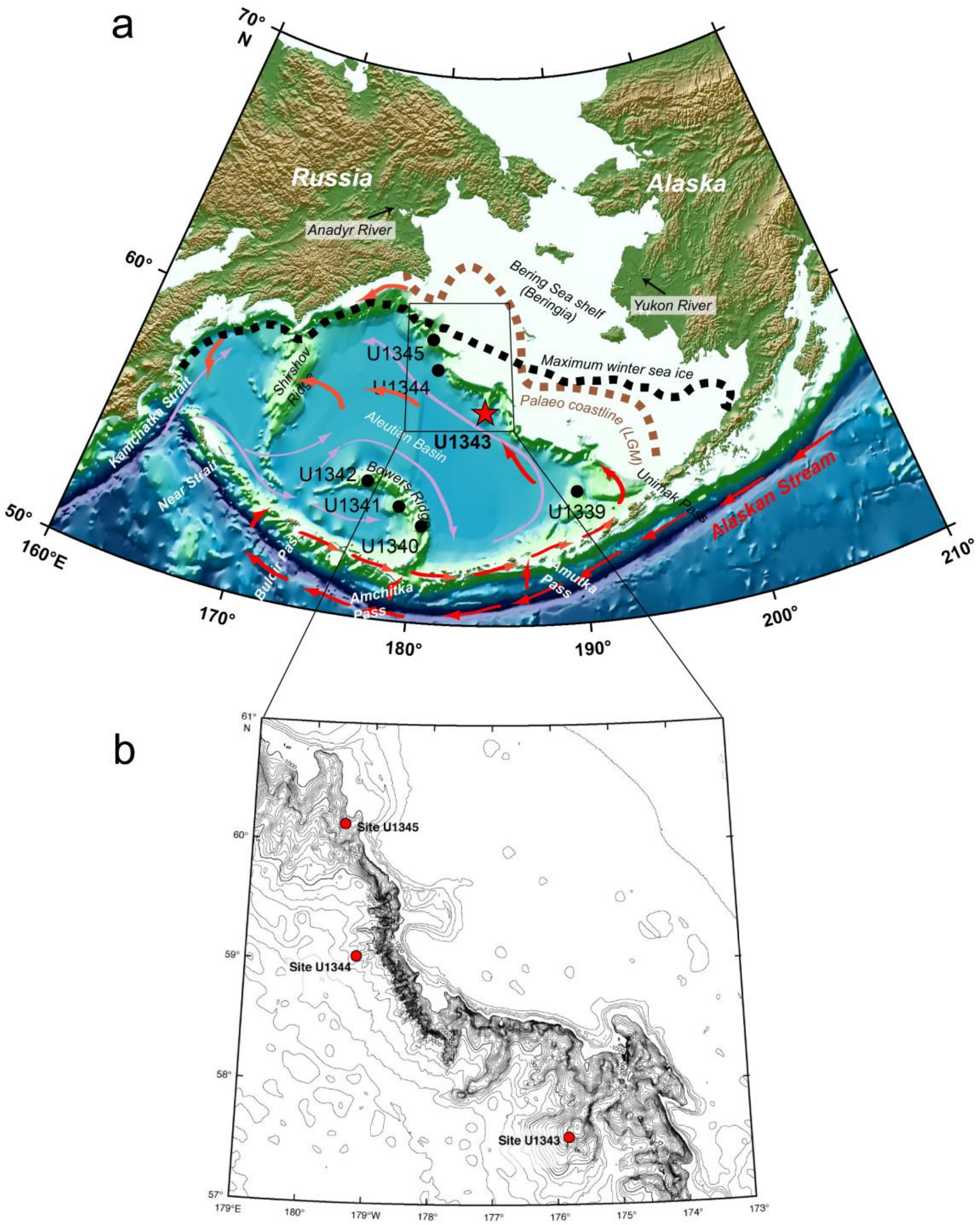

Supplementary Fig. 1. (a) Map of the Bering Sea and IODP Expedition 323 sites (star=U1343: this study), showing surface water currents (red arrows), deep water currents (purple arrows), maximum extent of winter sea ice (January climatology, >15% ice cover north of the line), and position of coastline during the last glacial maximum when sea level dropped ~120 m below present. **(b)** Detailed bathymetry of Sites U1343, U1344 and U1345. Note that the position of U1343 (this study) is on a topographic high. Maps adapted from Expedition 323 Scientists[1].

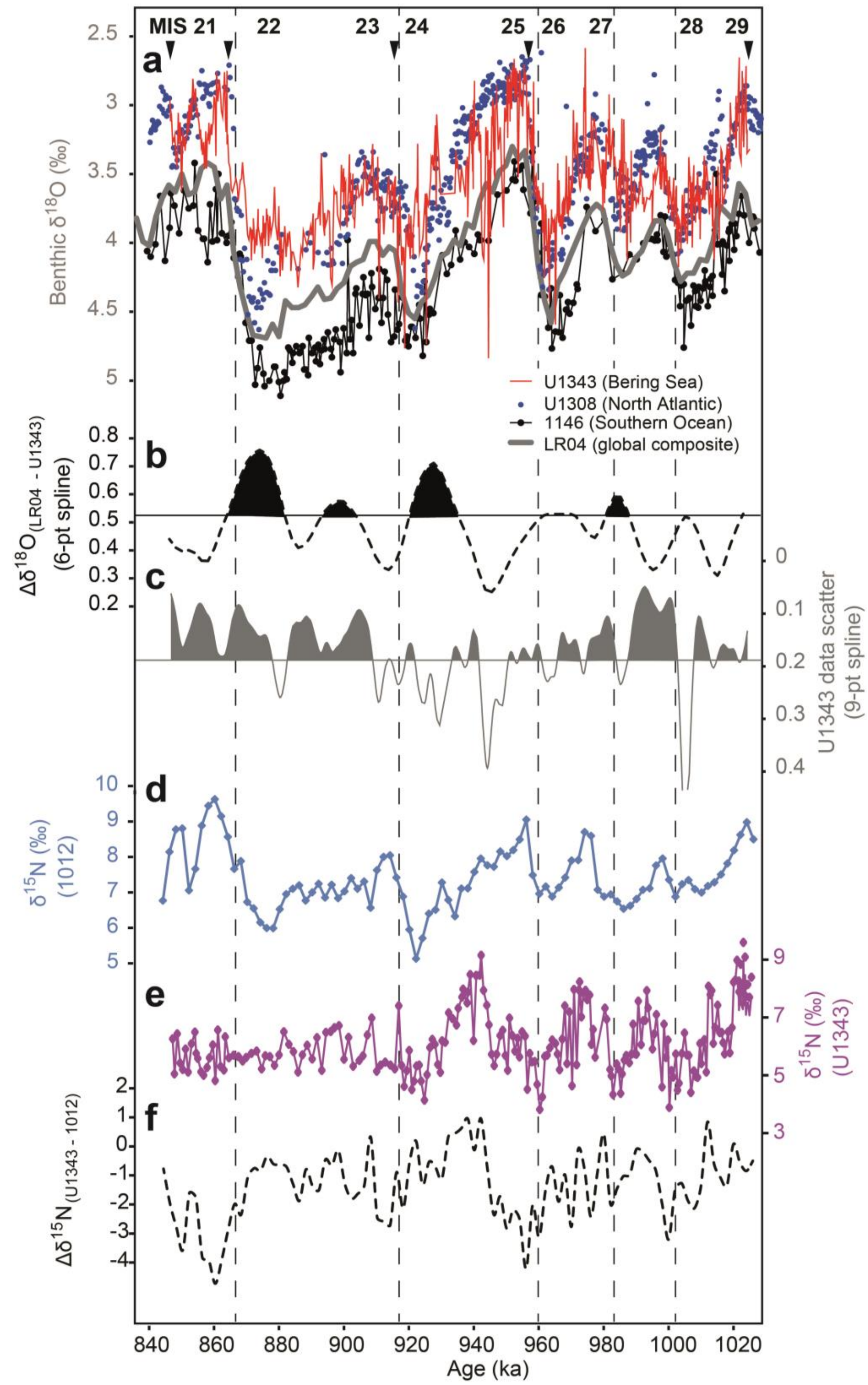

Supplementary Fig. 2. Foraminiferal oxygen isotope and bulk sedimentary nitrogen isotope data from Site U1343 compared with various published datasets. (a) Benthic $\delta^{18}O$ data for IODP Site U1343 (this study), with data from Site U1308[2], Site 1123[3] and LR04[4]. Note that all records apart from U1343 have a

pronounced $\delta^{18}O$ high at ~875 ka (MIS 22 glacial maximum). **(b)** Offset between U1343 and LR04 ($\Delta\delta^{18}O_{LR04\text{-}U1343}$) with a 6-pt smoothing spline. **(c)** Benthic $\delta^{18}O$ data scatter for U1343 (the difference between nearest neighbours) with a 9-pt smoothing spline (lower panel). **(d)** Bulk sediment $\delta^{15}N$ for northeast Pacific Site 1012[5]. **(e)** Bulk sediment $\delta^{15}N$ for Bering Sea Site U1343 (this study). **(f)** Bulk sediment $\delta^{15}N$ for Site 1012 subtracted from U1343 ($\Delta\delta^{15}N$) as a nutrient utilisation proxy for the Bering Sea. Black triangles are age control points between U1343 and LR04 benthic $\delta^{18}O$ records (this study).

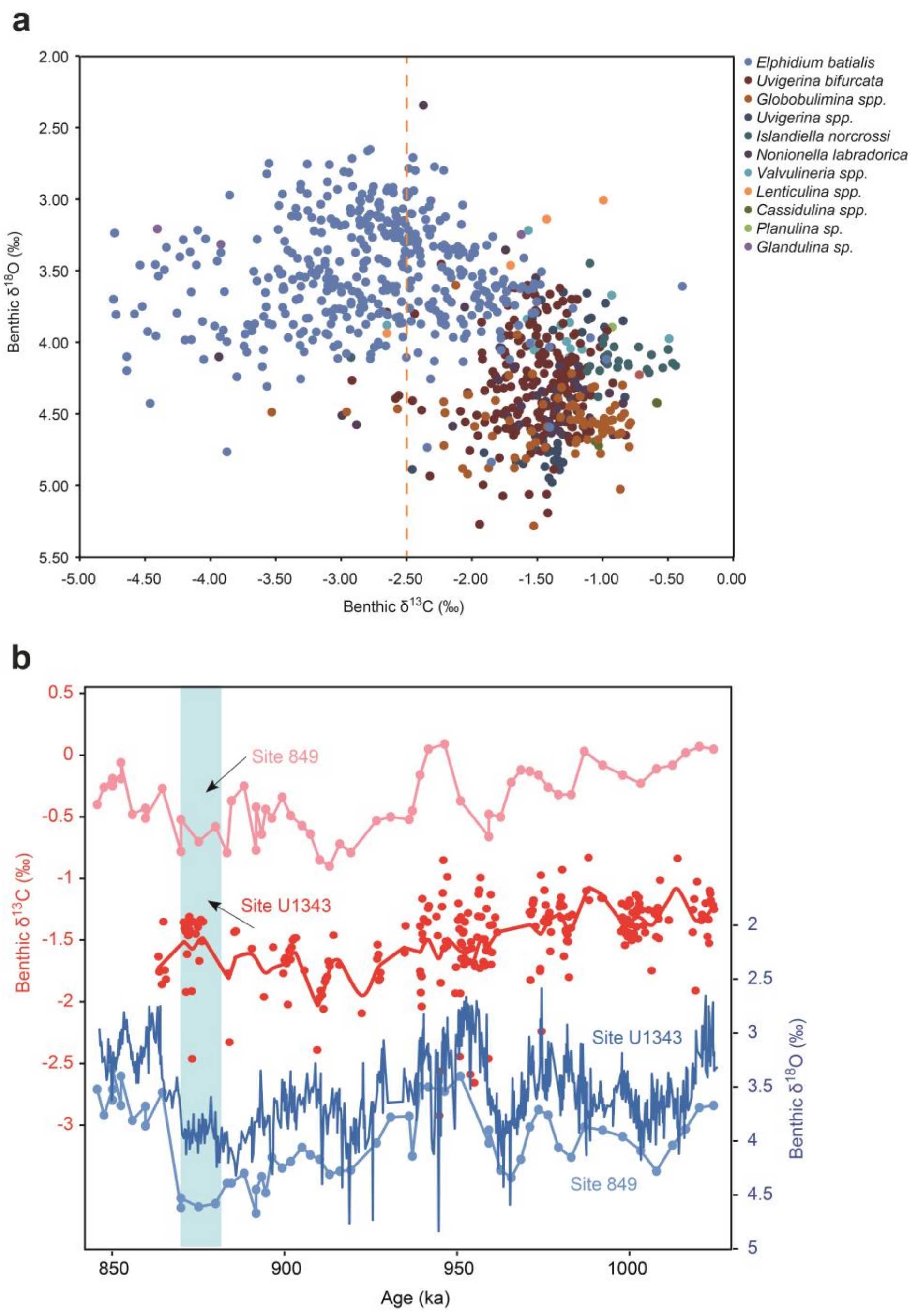

Supplementary Fig. 3. Foraminiferal carbon and oxygen isotope data from Site U1343 compared with published datasets. (a) Benthic foraminiferal $\delta^{18}O$ and $\delta^{13}C$ from various species at Site U1343 (this study). Samples with $\delta^{13}C$ values below –5 ‰ are not shown. **(b)** Benthic foraminiferal $\delta^{18}O$ (all species composite record) and $\delta^{13}C$ (*Uvigerina* spp. only) from Site U1343 (this study), along with $\delta^{18}O$ and $\delta^{13}C$ from equatorial East Pacific Site 849[6].

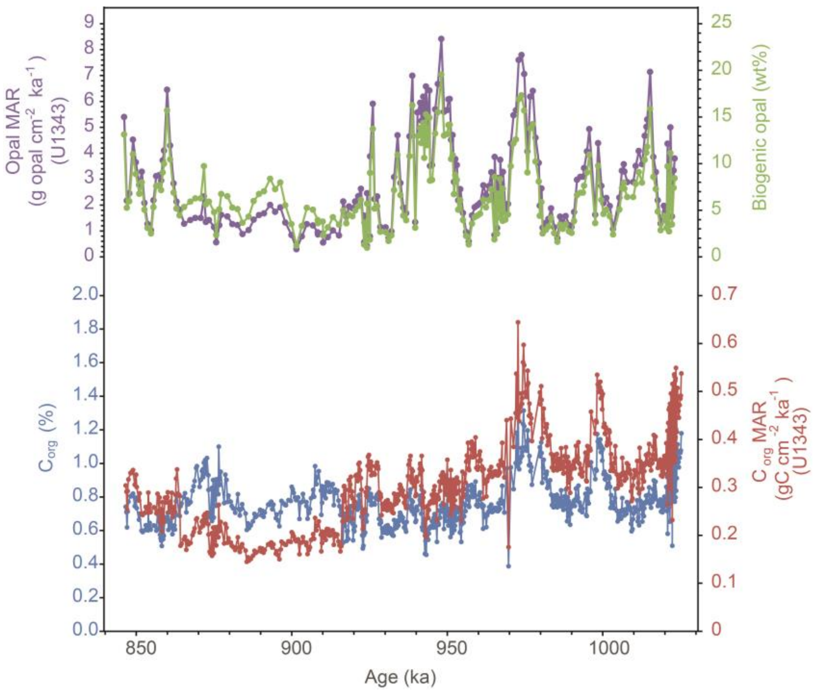

Supplementary Fig. 4. Comparison between % and MAR of proxies. Bulk sediment opal[7] and TOC (this study) are shown for Site U1343, both as % data and mass accumulation rates (MAR).

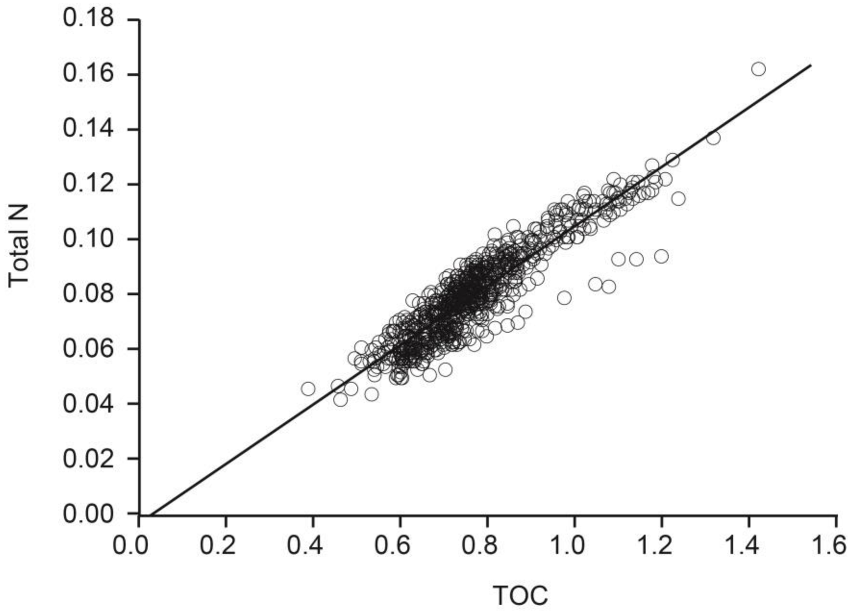

Supplementary Fig. 5. Total nitrogen (N %) against total organic carbon (TOC %) for Site U1343 (this study). Line represents a linear regression.

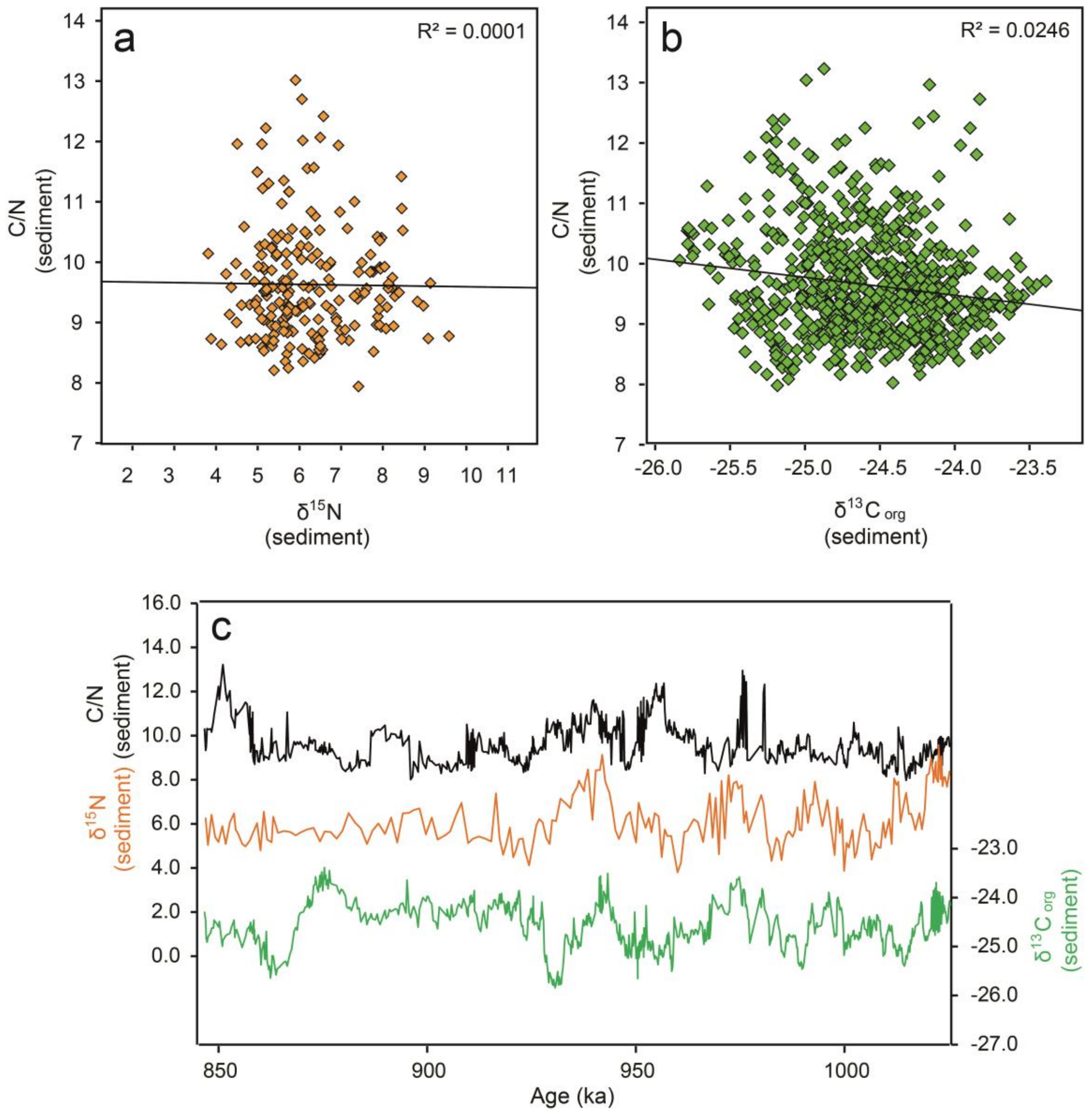

Supplementary Fig. 6. Bulk sediment C/N ratios against **(a)** $\delta^{15}N$ measured in the same samples, **(b)** $\delta^{13}C_{org}$ measured in the same samples, and **(c)** age. Linear regressions show no relationship between parameters.

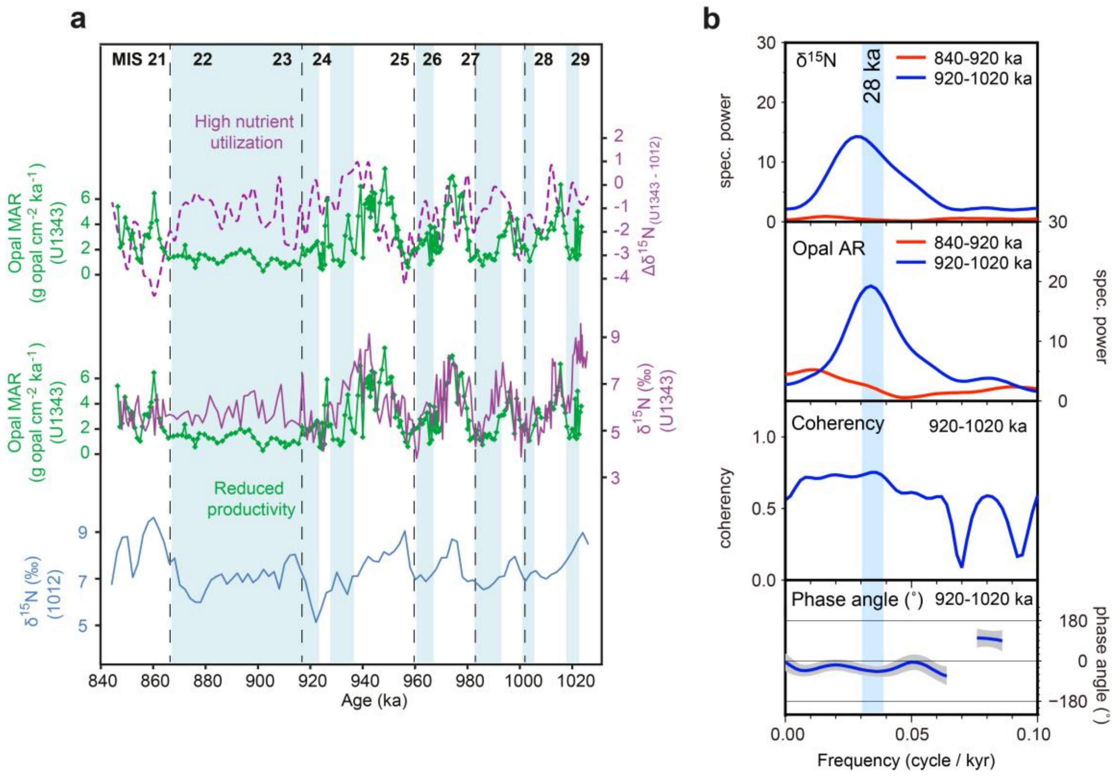

Supplementary Fig. 7. Analysis of the relationship between $\delta^{15}N$ and opal accumulation. **(a)** Bulk sediment $\delta^{15}N$ as a proxy for nutrient utilization for Site U1343 (this study), and Site 1012[5] subtracted from U1343 ($\Delta\delta^{15}N_{U1343-1012}$). Opal mass accumulation rate (MAR) as a proxy for productivity (green) for Site U1343[7]. **(b)** Spectral analysis (Blackman-Tukey cross spectrum, confidence level 80 %) of opal MAR and $\delta^{15}N$ for the time periods 920–840 ka (red) and 1020–920 ka (blue). No significant cyclic variations occur between 920–840 ka. Between 1020–920 ka both elevated spectral power and high coherency indicates significant cyclic variations are centred on 28 ka. Phase angle shows that opal MAR leads $\delta^{15}N$.

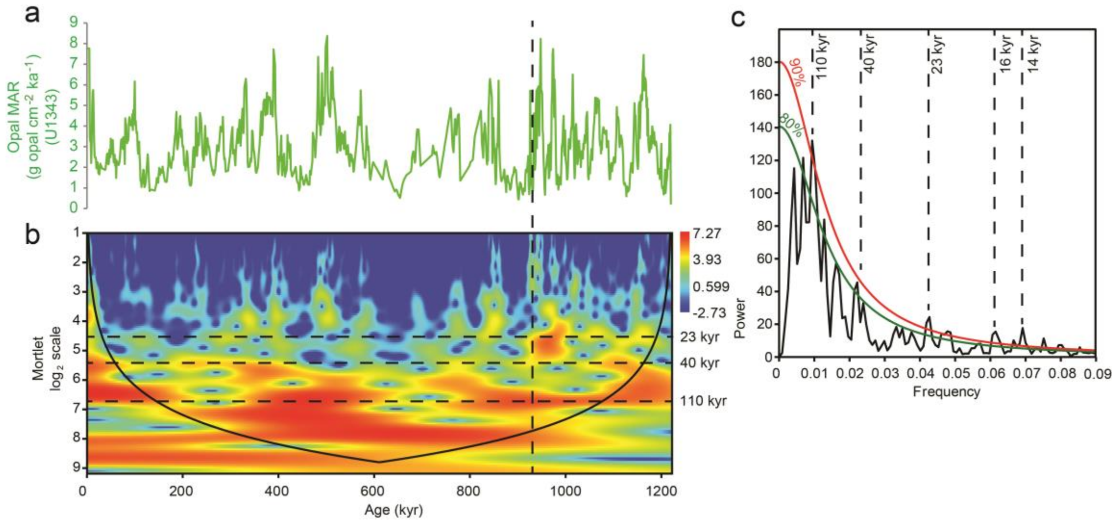

Supplementary Fig. 8. Data for IODP Site U1343, with spectra and wavelet analyses. **(a)** Bulk sediment wt% opal[7], converted to mass accumulation rate using modelled dry bulk density and published age model[8]. **(b)** Morlet wavelet power transform of interpolated (1 ka) opal data (with cone of influence). Note that significant 23 ka cyclicity occurs largely before ~900 ka. **(c)** Opal spectral analysis using REDFIT[9] (oversample 2, segments 3, rectangle window) of interpolated (1 ka) data.

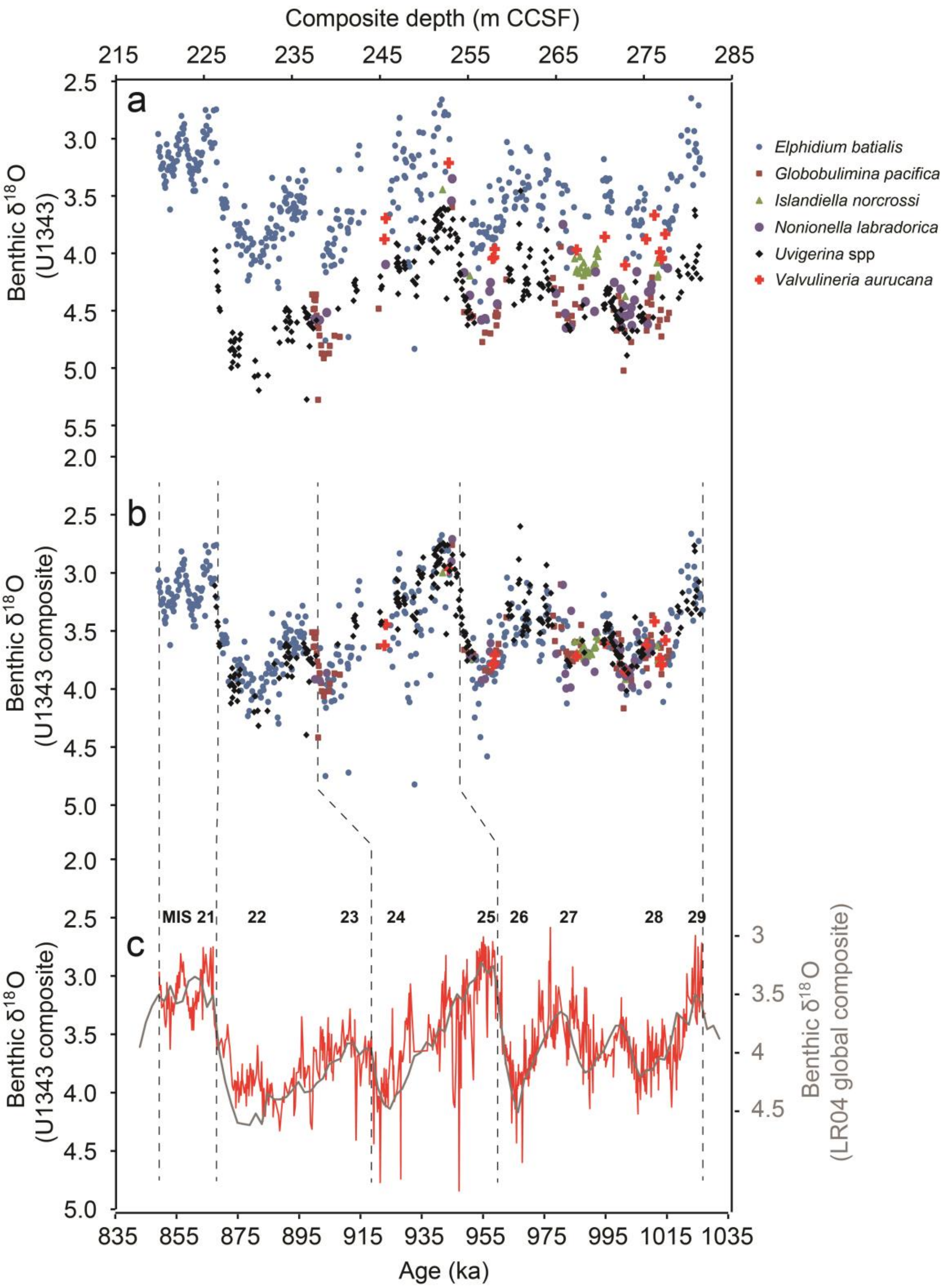

Supplementary Fig. 9. (a) Raw benthic foraminiferal δ^{18}O data from IODP Site U1343 from various species against depth. **(b)** Composite benthic δ^{18}O data from (a) with offsets applied (see Methods), against depth. **(c)** Composite benthic δ^{18}O against age, overlaid with LR04[4]. Tie points used to construct the age model are also shown.

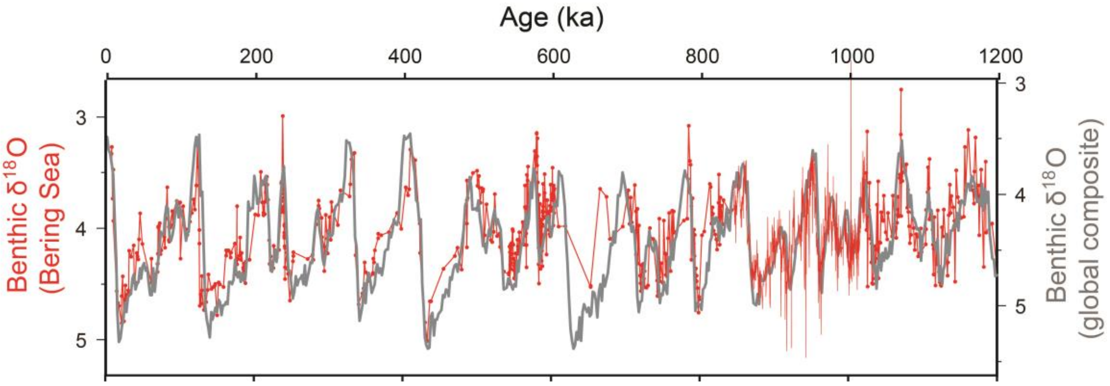

Supplementary Fig. 10. Benthic δ^{18}O data from Site U1343 against global composite stack LR04[4]. U1343 data between 840 and 1020 ka are from this study, other data from U1343 are from Asahi et al.[8].

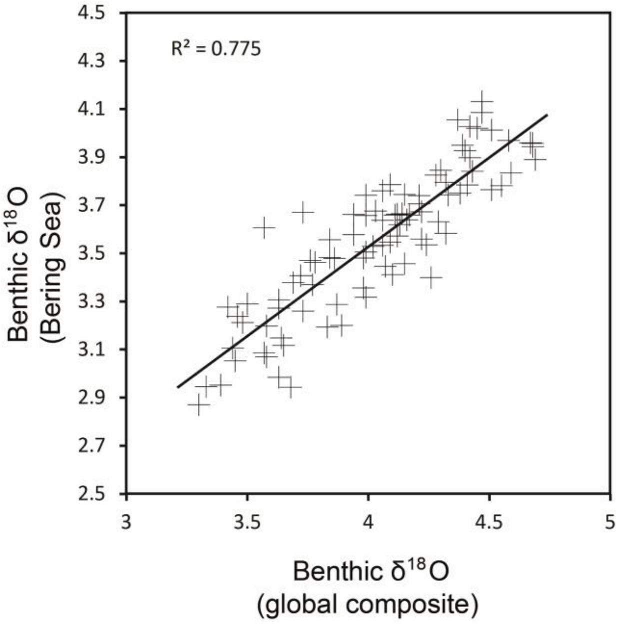

Supplementary Fig. 11. Benthic δ^{18}O data between 840–1020 ka from IODP Site U1343 (5-pt smoothing spline, linear interpolation), against LR04[4].

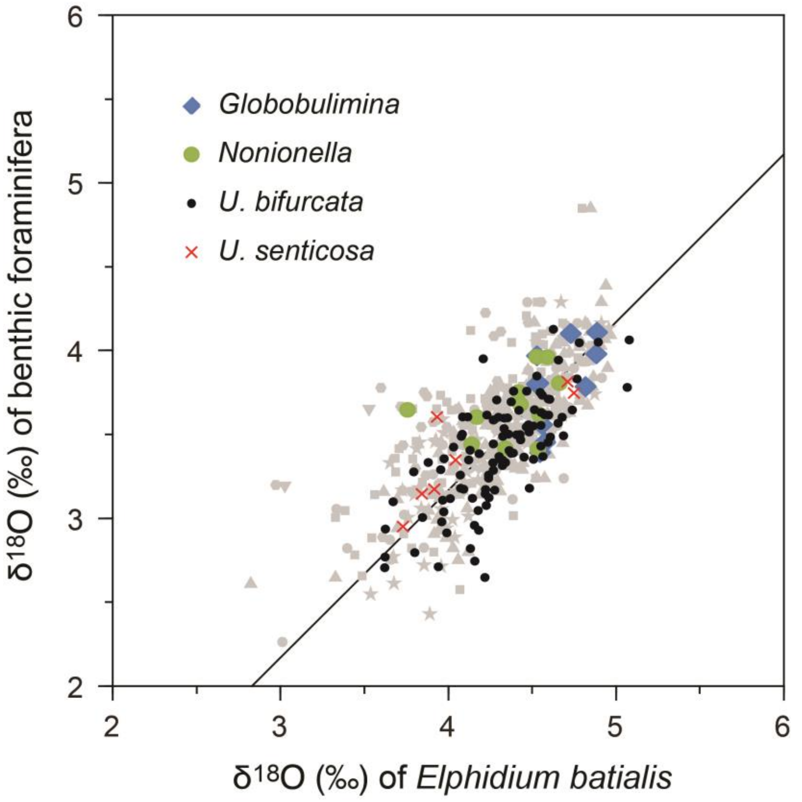

Supplementary Fig. 12. Various benthic foraminiferal species $\delta^{18}O$ from Site U1343 plotted against *Elphidium* $\delta^{18}O$ for values that were measured in the same sample. Grey markers are previously published data from Site U1343[4]. Line is 1:1.

Age (ka)	Composite depth (m CCSF)	Sedimentation rate (cm ka^{-1})
846.3	219.9	
864.2	226.5	37.36
916.3	237.9	21.78
956.5	253.6	39.13
1025.3	281.7	40.87

Supplementary Table 1. Age control points for correlating Site U1343 benthic $\delta^{18}O$ (this study) to global composite LR04[4]. A linear interpolation was assumed between points.

**Species**	**δ^{18}O offset to *E. batialis***	**(stdev)**	***n***
Uvigerina spp.	**0.87**	±0.20	208
Uvigerina senticosa	0.82	±0.22	61
Uvigerina bifurcata	0.89	±0.22	102
Uvigerina bifurcata	0.83	±0.25	53
Globobulimina pacifica	0.86	±0.21	10
Globobulimina pacifica	**0.85**	±0.22	147
*Nonionella labradorica**	0.62	±0.38	38
Nonionella labradorica	**0.65**	±0.27	91
*Islandiella norcrossi**	**0.48**	±0.08	25
*Valvulineria aurucana**	**0.25**	±0.12	15
Lenticulina spp.*	**-0.02**	±0.15	4
Cassidulina spp.*	**0.56**	±0.09	4

Supplementary Table 2. The mean offsets of the δ^{18}O of various species to *Elphidium batialis* from Site U1343. Offsets were calculated in samples where both species were measured, apart from species marked with * where offsets were calculated to the smoothing spline. Species underscored represent data from this study; other species are from previously published data from Site U1343[8]. Offsets in bold type are used to construct the final composite δ^{18}O record.

Supplementary references

1. Expedition 323 Scientists (2011) Site U1343. *Proceedings of the Integrated Ocean Drilling Program, 323,* eds Takahashi K, Ravelo AC, Alvarez Zarikian CA, and the Expedition 323 Scientists (Tokyo, IODP Management International, Inc.), doi:10.2204/iodp.proc.323.107.2011.
2. Hodell DA, Channell JET (2016) Mode transitions in Northern Hemisphere glaciation: co-evolution of millennial and orbital variability in Quaternary climate. *Clim. Past* 12:1805–1828.
3. Elderfield H, Ferretti P, Greaves M, Crowhurst S, McCave IN, Hodell D, Piotrowski, AM (2012) Evolution of ocean temperature and ice volume through the Mid-Pleistocene climate transition. *Science* 337:704–709.
4. Lisiecki LE, Raymo ME (2005) A Pliocene-Pleistocene stack of 57 globally distributed benthic δ18O records. *Paleoceanography* 20:PA1003.
5. Liu Z, Altabet MA, Herbert TD (2008) Plio-Pleistocene denitrification in the eastern tropical North Pacific: Intensification at 2.1 Ma. *Geochemistry Geophysics Geosystems* 9:11 doi: 10.1029/2008GC002044
6. Mix AC et al. (1995) Benthic foraminifer stable isotope record from Site 849 (0–5Ma): Local and global climate changes. *Proceedings of the Ocean Drilling Program, Scientific Results 138*, eds Pisias NG et al. (Ocean Drilling Program, College Station, Texas), pp 371–412.
7. Kim S, Takahashi K, Khim BK, Kanematsu Y, Asahi H, Ravelo AC (2014) Biogenic opal production changes during the mid-Pleistocene transition in the Bering Sea (IODP expedition 323 site U1343). *Quat. Res.* 81:151–157.
8. Asahi H, Kender S, Ikehara M, Sakamoto T, Takahashi K, Ravelo AC, Alvarez Zarikian CA, Khim BK, Leng MJ (2016) Orbital-scale benthic foraminiferal oxygen isotope stratigraphy at the northern Bering Sea Slope Site U1343 (IODP Expedition 323) and its Pleisocene paleoceanographic significance. *Deep Sea Res. II.* 125-126:66–83.
9. Schulz M, Mudelsee M (2002) REDFIT: estimating red-noise spectra directly from unevenly spaced paleoclimatic time series. *Comput. Geosci*. 28:421–426.
