# Supplementary material for: Closure of the Bering Strait caused Mid-Pleistocene Transition cooling
Source: Nat Commun. 2018 Dec 19;9:5386. doi: 10.1038/s41467-018-07828-0 (PMC6300599; doi:10.1038/s41467-018-07828-0)
Supplement: Supplementary file 2 — Description of Additional Supplementary Files [file 41467_2018_7828_MOESM2_ESM.pdf]

Supplementary Data 1.
Abundance of dinoflagellate cyst species and pollen and spore grains from IODP Site U1343.

Supplementary Data 2.
Oxygen and carbon isotopic values of benthic foraminifera from IODP Site U1343.

Supplementary Data 3.
Carbon and nitrogen percentage and isotope data of bulk sediment from IODP Site U1343.

Supplementary Data 4.
Nitrogen isotope data of bulk sediment from IODP Site U1343.
